# Invariant Natural Killer T Cell Agonist Modulates Experimental Focal and Segmental Glomerulosclerosis

**DOI:** 10.1371/journal.pone.0032454

**Published:** 2012-03-12

**Authors:** Rafael L. Pereira, Vanessa O. Reis, Patricia Semedo, Bruna N. Buscariollo, Cassiano Donizetti-Oliveira, Marcos A. Cenedeze, Maria Fernanda Soares, Alvaro Pacheco-Silva, Paul B. Savage, Niels O. S. Câmara, Alexandre C. Keller

**Affiliations:** 1 Departamento de Medicina – Nefrologia, Universidade Federal de São Paulo, São Paulo, Brasil; 2 Unidade de Transplante Renal, Instituto Israelita de Ensino e Pesquisa Albert Einstein, São Paulo, Brasil; 3 Department of Chemistry and Biochemistry Brigham Young University, Provo, Utah, United States of America; 4 Departamento de Imunologia, Universidade de São Paulo, São Paulo, Brasil; 5 Departamento de Microbiologia, Imunologia e Parasitologia, Universidade Federal de São Paulo, São Paulo, Brasil; Fondazione IRCCS Ospedale Maggiore Policlinico & Fondazione D'Amico per la Ricerca sulle Malattie Renali, Italy

## Abstract

A growing body of evidence demonstrates a correlation between Th2 cytokines and the development of focal and segmental glomerulosclerosis (FSGS). Therefore, we hypothesized that GSL-1, a monoglycosylceramide from *Sphingomonas ssp.* with pro-Th1 activity on invariant Natural Killer T (iNKT) lymphocytes, could counterbalance the Th2 profile and modulate glomerulosclerosis. Using an adriamycin(ADM)-based model of FSGS, we found that BALB/c mice presented albuminuria and glomerular degeneration in association with a Th2-like pro-fibrogenic profile; these mice also expressed a combination of inflammatory cytokines, such as IL-4, IL-1α, IL-1β, IL-17, TNF-α, and chemokines, such as RANTES and eotaxin. In addition, we observed a decrease in the mRNA levels of GD3 synthase, the enzyme responsible for GD3 metabolism, a glycolipid associated with podocyte physiology. GSL-1 treatment inhibited ADM-induced renal dysfunction and preserved kidney architecture, a phenomenon associated with the induction of a Th1-like response, increased levels of GD3 synthase transcripts and inhibition of pro-fibrotic transcripts and inflammatory cytokines. TGF-β analysis revealed increased levels of circulating protein and tissue transcripts in both ADM- and GSL-1-treated mice, suggesting that TGF-β could be associated with both FSGS pathology and iNKT-mediated immunosuppression; therefore, we analyzed the kidney expression of phosphorylated SMAD2/3 and SMAD7 proteins, molecules associated with the deleterious and protective effects of TGF-β, respectively. We found high levels of phosphoSMAD2/3 in ADM mice in contrast to the GSL-1 treated group in which SMAD7 expression increased. These data suggest that GSL-1 treatment modulates the downstream signaling of TGF-β through a renoprotective pathway. Finally, GSL-1 treatment at day 4, a period when proteinuria was already established, was still able to improve renal function, preserve renal structure and inhibit fibrogenic transcripts. In conclusion, our work demonstrates that the iNKT agonist GSL-1 modulates the pathogenesis of ADM-induced glomerulosclerosis and may provide an alternative approach to disease management.

## Introduction

Focal and segmental glomerulosclerosis (FSGS) is a growing cause of adult nephrotic syndrome and chronic kidney disease. Although FSGS presents diverse histological patterns and etiological associations, podocyte injury is a common denominator [1]. The immunological mechanisms involved in the pathogenesis of FSGS are not fully understood, but various studies demonstrate an association between a Th2-like profile and disease development. Yap and colleagues were the first to demonstrate a correlation between increased IL-13 mRNA expression and idiopathic nephrotic syndrome (INS) during childhood; because FSGS is one of the most common causes of INS, it was considered an indication of the association between Th2 cytokines and FSGS [2]. In the spontaneous FSGS Buffalo/Mna rat model, Le Berre and colleagues found an early imbalance in Th1/Th2 cytokines due to a T-cell infiltrate with a predominant Th2 profile, which in turn down-regulated Th1 responses [3]. Consistent with these results, Lai and colleagues demonstrated that IL-13 over-expression induced minimum change-like nephropathy, a phenomenon associated with podocyte structural changes and increased expression of IL-4Rα and IL-13Rα2 in the glomeruli [4].

Combined, these previous studies support a correlation between Th2 cytokines and the development of FSGS. Because of the antagonism between Th1 and Th2 cytokines, we hypothesized that the polarization of immune responses toward a Th1 profile could inhibit or even modulate the pathogenesis of FSGS. In this sense, the activation of invariant natural killer T lymphocytes (iNKT) by their agonist α-galactosylceramide (α-GaCer) or analogs has been shown to increase Th1-mediated responses, a property that has been used successfully to modulate Th2-mediated diseases, such as asthma [5], [6], [7], [8].

iNKT cells are non-conventional lymphocytes that can modulate the outcome of different immune-mediated diseases through the prompt secretion of different cytokines upon TCR stimulation [9]. A characteristic feature of iNKT cells is their selectivity for glycolipid antigens presented by the nonpolymorphic MHC class I-like molecule CD1d, which has been used to modulate different immune responses by exogenous agonists [10], [11], [12]. We chose to study the effect of GSL-1, a monoglycosylceramide obtained from *Sphingomonas ssp.* with a pro-Th1 nature, on FSGS pathogenesis [13]. To this end, we used an experimental model that is based on the susceptibility of podocytes to the cytotoxic effects of doxorubicin hydrochloride, also known as adriamycin (ADM) [14].

ADM-induced FSGS not only has an immune system-dependent component but is also a reliable mimetic of the human disease [15], [16]. Although the immune component is not fully understood, Th2-prone strains, such as BALB/c mice, are well known to be more susceptible to ADM injury, corroborating the idea that FSGS is a Th2-associated disease. Therefore, ADM-FSGS is a useful model to both reproduce the disease and study the effects of Th1 polarization on disease pathogenesis.

In this study, we demonstrate that GSL-1 treatment modulates the development of ADM-induced FSGS in an iNKT-dependent manner. A Th1-polarization, increased mRNA for enzymes associated with ganglioside metabolism, and the modulation of proteins involved with the regulation of TGF-β downstream signaling were observed in this model.

## Results

### GSL-1 treatment inhibits ADM-induced proteinuria and albuminuria

**Figure 1:**
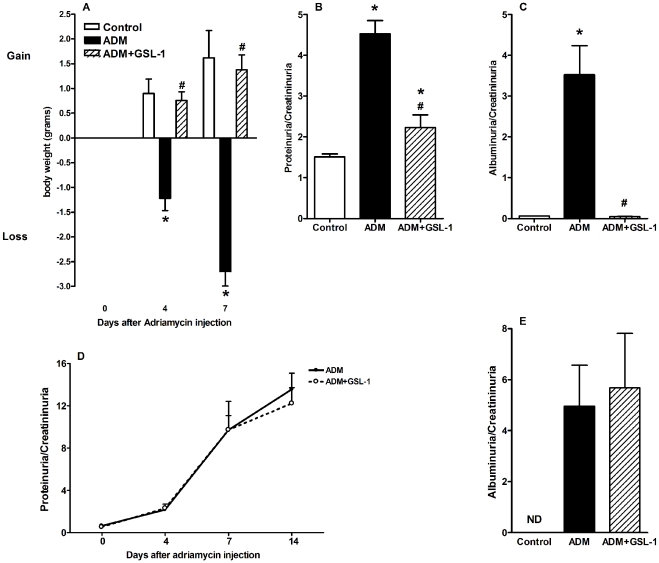
GSL-1 treatment inhibits ADM-induced renal failure due to podocyte injury in an iNKT-dependent manner. BALB/c WT or iNKT-deficient (Jalpha18^−/−^) mice were injected at day 0 with 10 mg/kg of adriamycin (ADM) or treated concomitantly with ADM and 5 µg/mouse GSL-1 (ADM+GSL-1). (A) As a result of ADM cytotoxicity, the mice lost body weight in a time-dependent fashion, in contrast to the control, untreated mice, and the GSL-1-treated group. (B) The increased proteinuria/creatininuria found in the ADM group at day 7 post-ADM injection reflects impaired renal function compared with the control and ADM+GSL-1 animals. These data indicate a renoprotective effect of GSL-1 treatment. (C) The albuminuria levels found in ADM animals reflect the podocyte injury due to ADM cytotoxicity. In contrast, the albuminuria/creatininuria ratio in the ADM+GSL-1 group was comparable to that in the control animals, indicating podocyte conservation. The GSL-1 treatment failed to protect Jalpha18^−/−^ mice from ADM-induced renal injury, showing that its renoprotective effect is iNKT-dependent (D and E). * p<0.05 vs. control; # p<0.05 vs. ADM.

We first tested the effect of GSL-1 treatment on the renal failure induced by ADM administration. BALB/c mice, aged 8 to 10 weeks, were injected at day 0 with ADM (10 mg/Kg) or ADM plus GSL-1 (5 µg/animal). Figure 1A demonstrates that ADM mice lost body weight in a time-dependent manner, likely as a result of ADM-induced nephropathy. Consistent with this result, the proteinuria/creatininuria ratio at day 7 was significantly elevated in the ADM group compared with the control mice, indicating impaired renal function (Figure 1B). In contrast, GSL-1-treated mice gained body weight, as did the control animals, suggesting that GSL-1 administration exerts a protective effect on ADM-induced disease. In fact, the proteinuria levels found in ADM+GSL-1 mice were comparable to that of the control group, corroborating the renoprotective effect of GSL-1 treatment. The total proteinuria/creatininuria ratio can be a marker for renal function but is not a reliable indicator of glomerular alterations. In contrast, albumin is a high molecular weight protein that is not found in urine, and therefore, an increase in albumin levels reflects a loss of the glomerular filtration barrier (GFB) due to podocyte injury. Therefore, we measured the albuminuria/creatininuria ratio to better characterize the extent of podocyte injury. Consistent with data from Figure 1B, ADM mice showed increased albuminuria compared with control and ADM+GSL-1 mice, supporting the idea that GSL-1 treatment protected podocytes from ADM cytotoxicity and consequently preserved the GFB (Figure 1C).

To better address the relationship between the renoprotective effects of GSL-1 treatment and iNKT activation, we took advantage of the BALB/c iNKT-deficient (Jalpha18^−/−^) strain [17]. As in BALB/c WT mice, ADM administration in Jalpha18^−/−^ animals resulted in a time-dependent loss of renal function, illustrated by the increase in the proteinuria/creatininuria ratio (Figure 1D). The glomerular damage was represented by the elevated levels of albuminuria found at day 28 post-ADM (Figure 1E). In contrast with the WT mice, the treatment of Jalpha18^−/−^ mice with GSL-1 was unable to inhibit FSGS development; therefore, our data demonstrate that the protective effect of GSL-1 is iNKT-dependent, corroborating the specificity of GSL-1 as an iNKT agonist.

### GSL-1 treatment inhibits ADM-induced renal injury

**Figure 2:**
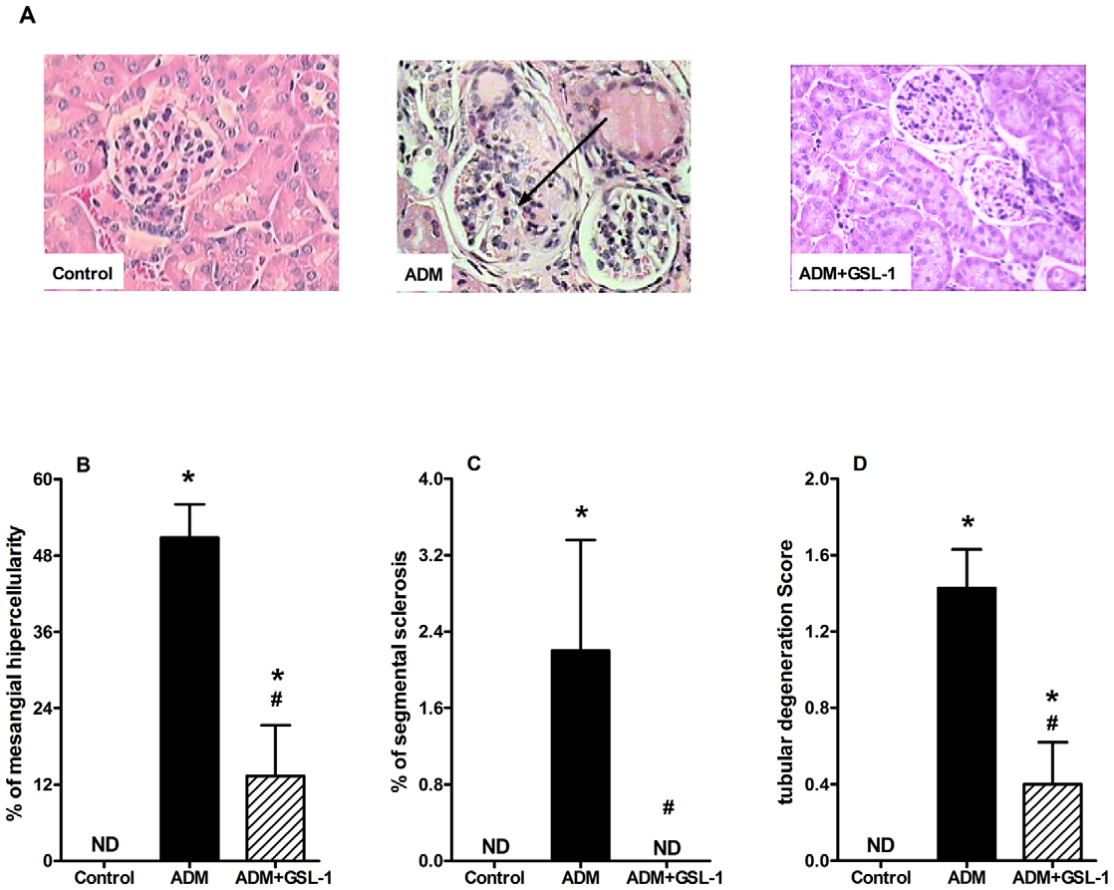
GSL-1 treatment inhibits renal injury due to ADM cytotoxicity. BALB/c mice were injected at day 0 with 10 mg/kg of adriamycin (ADM) or treated concomitantly with ADM and 5 µg/mouse GSL-1 (ADM+GSL-1). (A) Representative image of the renal alterations induced by ADM administration (black arrow). Figures B to D show graphic representation of the mesangial hypercellularity, signs of glomerular sclerosis and tubular degeneration induced by ADM administration. Figure 2 corroborates the renoprotective effect of GSL-1 treatment depicted in Figure 1. * p<0.05 vs. control; # p<0.05 vs. ADM.

To determine the extent of the renal tissue damage, we performed histological analyses at day 7 post-ADM. Figure 2 demonstrates that ADM injection resulted in alterations of renal tissue, inducing mesangial hypercellularity, signs of glomerular sclerosis and tubular degeneration when compared with control mice (Figures 2A to 2D, respectively). In contrast, GSL-1 treatment preserved renal architecture, corroborating the conservation of renal function depicted in Figure 1.

### GSL-1 treatment modulates the pro-Th2 milieu induced by ADM and inhibits the expression of pro-fibrotic transcripts

**Figure 3:**
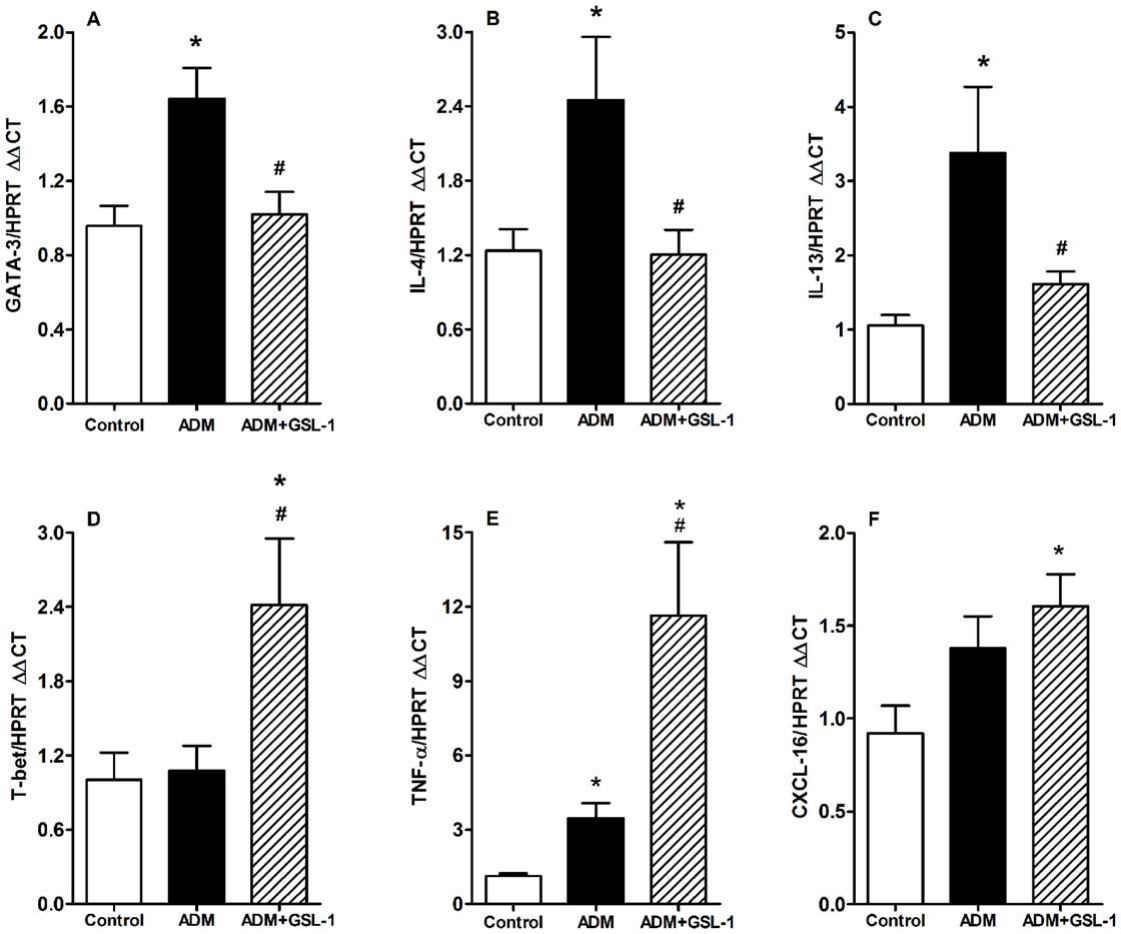
GSL-1 treatment modulates the ADM-induced Th2-like cytokine profile and induces a pro-Th1 environment. BALB/c mice were injected at day 0 with 10 mg/kg of adriamycin (ADM) or treated concomitantly with ADM and 5 µg/mouse GSL-1 (ADM+GSL-1). The ADM mice had increased levels of mRNA for the Th2-related transcription factor GATA3 (A), reflecting increased transcript levels for IL-4 (B) and IL-13 (C). In contrast, GSL-1 treatment inhibited these transcripts in association with an increase in the levels of mRNA for T-bet (D), a transcription factor associated with Th1 responses, TNFα (E) and the chemokine ligand CXCL16 (F). Thus, these data corroborate the idea that ADM-induced FSGS is associated with a Th2-like profile that can be inhibited by GSL-1 treatment and the generation of a pro-Th1 environment. * p<0.05 vs. control; # p<0.05 vs. ADM.

The qPCR analyses of renal tissue revealed that ADM injection resulted in an early increase in the mRNA levels of the pro-Th2 transcription factor GATA3 along with transcripts for IL-4 and IL-13, two cytokines extensively associated with Th2 responses (Figures 3A to 3C, respectively) [18], [19], [20]. In contrast, GSL-1 treatment inhibited GATA3 and IL-4 mRNA expression, a phenomenon associated with increased mRNA expression of T-bet (T box expressed in T-cells), a pro-Th1-related transcription factor, TNF-α, a classical type 1 cytokine and CXCL16, a chemokine associated with iNKT-mediated Th1 responses (Figures 3D to 3F, respectively) [21], [22]. These data are consistent with the idea that FSGS is associated with Th2 responses and support the hypothesis that GSL-1 treatment promotes a pro-Th1 polarization of the immune system and thereby protects the kidneys.

**Figure 4:**
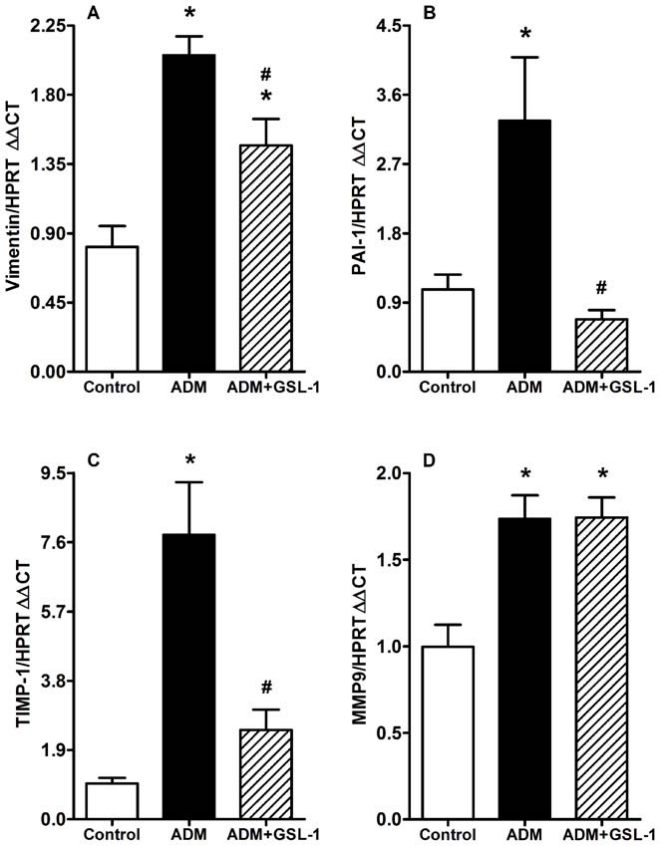
GSL-1 treatment inhibits the ADM-induced expression of fibrogenic mRNAs. BALB/c mice were injected at day 0 with 10 mg/kg of adriamycin (ADM) or treated concomitantly with ADM and 5 µg/mouse GSL-1 (ADM+GSL-1). (A) ADM mice presented increased levels of vimentin compared with control mice; vimentin was diminished in GSL-1-treated group. (B) The levels of PAI-1 transcripts significantly increased in ADM mice, in contrast with ADM+GSL-1 animals, which had levels comparable to that of the control mice. (C) TIMP-1 mRNA increased in the ADM group compared with the control and ADM+GSL-1 mice (D) MMP9 transcripts were equally increased in the ADM and ADM+GSL-1 groups compared with the control mice. These data indicate the development of ADM-induced FSGS with renal remodeling. * p<0.05 vs. control; # p<0.05 vs. ADM.

Because FSGS pathology can be associated with kidney fibrosis, we decided to determine the mRNA expression of vimentin, plasminogen activator inhibitor-1 (PAI-1) and TIMP-1 (tissue inhibitor of metalloproteinase-1). Figure 4A demonstrates that ADM mice showed increased levels of mRNA for vimentin, which was diminished in the GSL-1 group. Consistent with these results, we found that the induction of PAI-1 mRNA was inhibited in GSL-1 treated mice compared with the ADM group (Figure 4B). Finally, TIMP-1 transcript levels also increased in the ADM group compared with control and ADM+GSL-1 mice (Figure 4C). The mRNA levels of metalloproteinase 9 (MMP9) were slightly increased in both ADM and ADM+GSL-1 animals when compared with the control group (Figure 4D). The western blot analysis of kidney tissue corroborated the MMP9 and PAI-1 transcript findings, also showing a significant increase in desmin in ADM animals compared with both control and ADM+GSL-1 animals (Figure S1). This result corroborates previous findings associating podocyte injury with desmin expression [23]. Taken together, our data demonstrate that the renoprotective effect of GSL-1 is associated with the inhibition of important fibrogenic-associated factors that are increased in response to ADM-induced injury.

### GSL-1 treatment inhibits the inflammatory context induced by ADM

**Figure 5:**
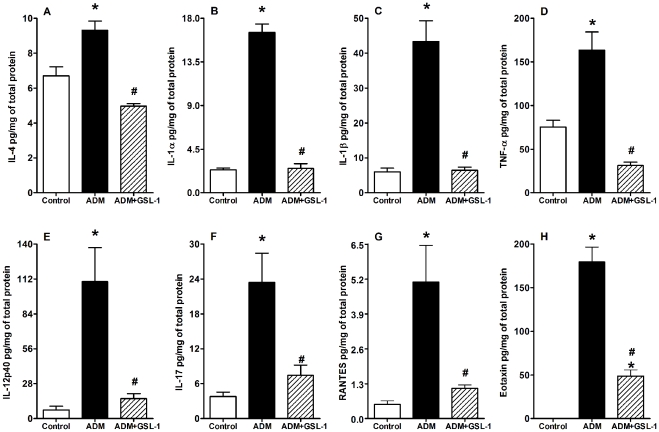
GSL-1 treatment inhibits the inflammatory context induced by ADM administration. BALB/c mice were injected at day 0 with 10 mg/kg of adriamycin (ADM) or treated concomitantly with ADM and 5 µg/mouse GSL-1 (ADM+GSL-1). ADM mice had increased levels of the inflammatory cytokines IL-4, IL-1α, IL-1β, TNF-α, IL-12p40 and IL-17 compared with both the control and GSL-1-treated groups (A to F, respectively). The administration of ADM also induced high levels of inflammatory chemokines, such as RANTES and eotaxin, which were inhibited by GSL-1 treatment (G and H, respectively). These data indicate an inhibitory effect for GSL-1 treatment, supporting its renoprotective effect. * p<0.05 vs. control; # p<0.05 vs. ADM.

To better characterize the inflammatory environment associated with ADM-induced FSGS pathogenesis, we performed a multiparameter analysis of inflammatory cytokines present in kidney tissue. Figure 5 demonstrates increased levels of IL-4, IL-1α, IL-1β, TNF-α, IL-12p40 and IL-17 in the ADM group compared with the control animals; such increases were not observed in GSL-1-treated mice (Figures 5A, 5B, 5C, 5D, 5E and 5F, respectively). In addition, we found high levels of RANTES and eotaxin in ADM- but not in GSL-1-treated mice (Figures 5G and H, respectively); eotaxin is a chemokine extensively associated with Th2 inflammation [24]. These data demonstrate that, together with a Th2-like profile, FSGS development is also associated with cytokines from the innate and Th17 “arms” of the immune response. Moreover, these results further demonstrate the inhibitory effect of GSL-1 treatment on the inflammatory response induced by ADM, supporting its renoprotective effect.

### GSL-1 treatment induces an alternative TGF-β signaling pathway

**Figure 6:**
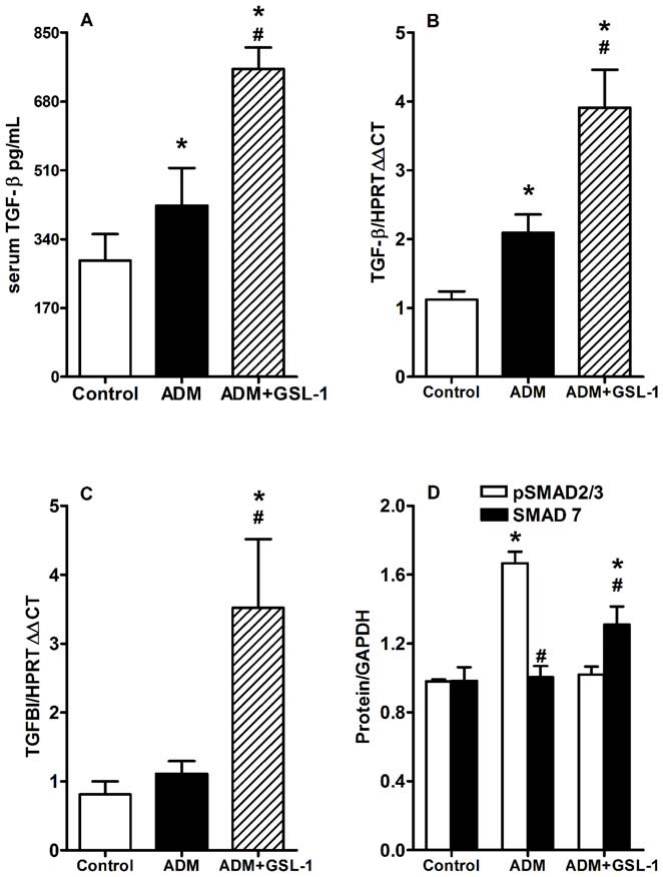
GSL-1 treatment modulates TGF-β signaling. BALB/c mice were injected at day 0 with 10 mg/kg of adriamycin (ADM) or treated concomitantly with ADM and 5 µg/mouse GSL-1 (ADM+GSL-1). (A) Total TGF-β protein increased in the serum of both the ADM and ADM+GSL-1 groups compared with the control animals. (B) The increase in TGF-β transcripts in kidney tissue reflected the serum findings showing that the ADM and ADM+GSL-1 groups had higher levels of this cytokine compared with the control mice. GSL-1 treatment induced a more significant increase in serum TGFβ and mRNA transcripts compared with the ADM group; however, only ADM+GSL-1 mice presented transcripts to the TGFBI/BIGH3 protein (C), indicating that TGF-β production had a different effect in this group. This idea is supported by the presence of phosphorylated SMAD2/3 in ADM mice, whereas in the ADM+GSL-1 group, the expression of SMAD7 protein increased (D). These data indicate that GSL-1 treatment modulated the downstream cascade of TGF-β signaling. * p<0.05 vs. control; # p<0.05 vs. ADM.

Glomerulosclerosis pathogenesis has been extensively associated with TGF-β synthesis and/or signaling; therefore, we decided to determine the effect of GSL-1 on TGF-β production. Figure 6A demonstrates that ADM injection increased the serum levels of total TGF-β when compared with control mice, reinforcing the idea that TGF-β overproduction is associated with glomerulosclerosis [25]. Unexpectedly, we found that GSL-1 treatment further elevated the serum levels of TGF-β. qPCR analysis of kidney tissue revealed that the induction of TGF-β mRNA exactly reflected the serum findings. The ADM group showed higher levels of TGF-β transcripts than the control animals but lower than those found in GSL-1 mice (Figure 6B). These data suggest that, in our model, TGF-β could be associated with either a renoprotective or deleterious effect depending on the context in which it is produced.

We have previously shown that a renoprotective TGF-β signal was associated with iNKT cells and the induction of TGF-β-induced gene (TGFBI/BIGH3) transcripts [26]. Further PCR analysis revealed that only treatment with GSL-1 was able to induce the transcription of TGFBI mRNA (Figure 6C), suggesting that the activation of iNKT cells by pro-Th1 agonists induced an immunological *milieu* that favors the renoprotective effect of TGF-β. To better understand this phenomenon, we used western blotting to analyze the protein expression of SMAD2/3 and SMAD7, which have been extensively associated with TGF-β signaling. Figure 6D shows that ADM injection was associated with increased levels of phosphorylated SMAD2/3 protein, whereas GSL-1 treatment induced a slight increase in the levels of SMAD7 protein. These data reinforce the idea that the effect of TGF-β is dependent on the immune context induced by ADM or ADM+GSL-1 treatment.

### GSL-1 treatment increases the levels of GM3 and GD3 ganglioside synthase transcripts

**Figure 7:**
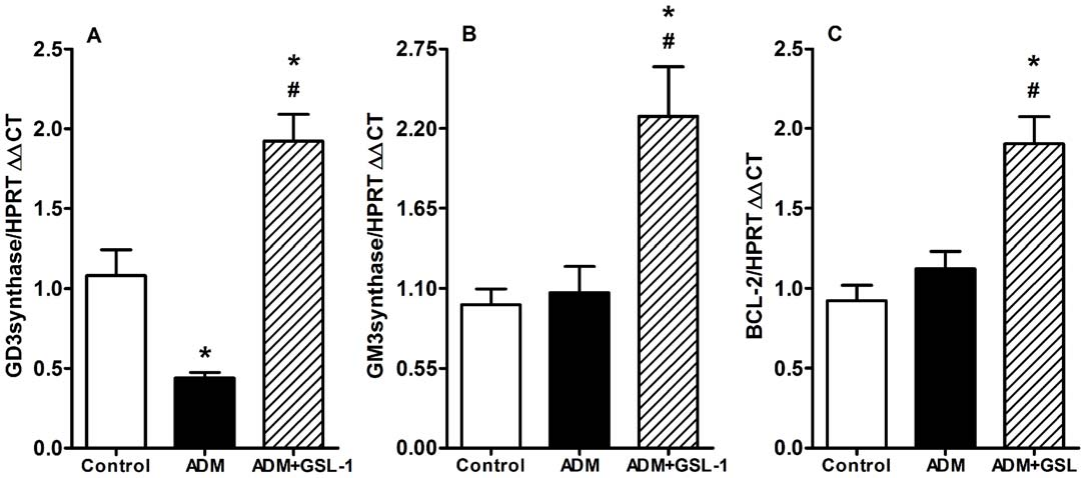
GSL-1 treatment induces transcripts for GM3 and GD3 synthases and the anti-apoptotic protein Bcl-2. BALB/c mice were injected at day 0 with 10 mg/kg of adriamycin (ADM) or treated concomitantly with ADM and 5 µg/mouse GSL-1 (ADM+GSL-1). (A) The ADM group showed decreased levels of ST8Sia1, the enzyme responsible for the generation of GD3 gangliosides, molecules that have been associated with podocyte physiology. In contrast, GSL-1 treatment increased the levels of this transcript compared with both control and ADM mice. (B) The levels of mRNA for GM3 synthase (ST3Gal5) were not altered in the ADM group; however, they were significantly augmented after GSL-1 treatment. Because GM3 gangliosides were associated with resistance to ADM-cytotoxicity due to Bcl-2 induction, we measured the mRNA levels of this protein in our system. (C) Bcl-2 transcripts were not altered by ADM but increased in ADM+GSL-1 mice, indicating a relationship between increased ST3Galt5 and Bcl-2 transcripts with renal protection from effects of ADM. * p<0.05 vs. control; # p<0.05 vs. ADM.

ADM-induced podocyte injury has been associated with decreased levels of glomerular GD3 ganglioside, whereas iNKT cell activation has been associated with the synthesis of glycolipids, predominantly gangliosides [27], [28]. Therefore, we determined the mRNA expression of different classes of enzymes involved in glycolipid metabolism. We found that ADM mice showed a significant decrease in mRNA levels for GD3 synthase (ST8Sia1), whereas GSL-1-treated mice showed increased expression of ST8Sia1 transcripts (Figure 7A). These results are consistent with previous studies showing a correlation between GD3 gangliosides and kidney physiology [29], [30], [31], [32]. In addition, the expression of GM3 gangliosides has been shown to be associated with protection from ADM-cytotoxicity through the induction of the anti-apoptotic protein Bcl-2 [33]. Although ADM administration did not influence the levels of GM3 synthase (ST3Gal5) transcripts, GSL-1 treatment induced a significant increase in the mRNA levels of this enzyme (Figure 7B). Consistent with the idea that GM3 could be associated with renoprotection through the induction of Bcl-2, we detected increased levels of Bcl-2 transcripts in ADM+GSL-1 mice (Figure 7C). Taken together, these data suggest that iNKT activation due to GSL-1 administration induces endogenous glycolipids that can, in turn, protect podocytes from ADM cytotoxicity.

### GSL-1 treatment ameliorates ADM-induced FSGS

**Figure 8:**
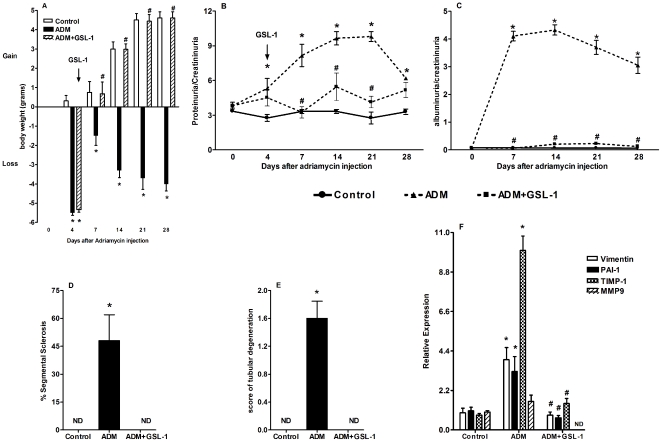
GSL-1 treatment reversed the time-dependent ADM-induced renal failure. BALB/c mice were injected at day 0 with 10 mg/kg of adriamycin (ADM) or treated with ADM and four days later with 5 µg/mouse GSL-1 (ADM+GSL-1). (A) The loss of body weight associated with ADM injection was reversed after GSL-1 treatment. The time-dependent renal failure, characterized by increases in the proteinuria (B) and albuminuria (C) ratios, was inhibited after GSL-1 treatment at day 4 post-ADM injection. This amelioration in renal function was associated with kidney preservation (D and E) and the inhibition of transcripts for fibrogenic proteins (F). * p<0.05 vs. control; # p<0.05 vs. ADM.

Finally, we decided to determine whether the renoprotective effect of GSL-1 could be extended to the treatment of an ongoing disease. Therefore, we initiated GSL-1 treatment at day 4 post-ADM injection, a time point when proteinuria was already elevated when compared with control animals. Figure 8A demonstrates that the time-dependent loss of body weight induced by ADM administration was reversed by GSL-1 treatment at day 4. This phenomenon was associated with the reversion of time-dependent renal failure, indicated by a continuous increase in proteinuria and albuminuria levels in the ADM mice (Figures 8B and 8C, respectively). Figures 8D and 8E demonstrate that the recovery of renal function was associated with kidney preservation. The transcripts for vimentin, PAI-1 and TIMP-1 were also elevated in the ADM group when compared with the GSL-1 treated mice. Moreover, MMP9 mRNA was not detected in ADM+GSL-1 mice, suggesting that MMP9 transcription was inhibited (Figure 8F). Therefore, our data demonstrate that GSL-1 administration during the early stage of disease can reverse its pathogenesis.

## Discussion

The mechanisms involved in FSGS pathogenesis are still not understood, but different studies have demonstrated, in both humans and experimental models, that this disease is associated with increased levels of Th2 cytokines [2], [3], [4]. Therefore, we first hypothesized that GSL-1, a pro-Th1 iNKT agonist, could reverse the Th2-like profile associated with FSGS and thereby modulate disease pathology. In fact, GSL-1 treatment inhibited the inflammatory context induced by ADM and preserved renal function, even when administered after proteinuria had already been established.

Regardless of the diverse etiology and histological variations of FSGS, it is clear that podocyte injury plays a pivotal role in this disease. To test the hypothesis that GSL-1 could inhibit Th2 responses and thereby modulate glomerulosclerosis, we used the ADM-induced FSGS model, an experimental model based on podocyte susceptibility to doxorubicin cytotoxicity. As discussed by Lee and Harris, this susceptibility depends on the immunological tendency of the strain, illustrated by the difference in susceptibility between BALB/c and C57BL/6 mice, which are described as Th2- and Th1-prone strains, respectively [34]. It is well documented that BALB/c mice are more susceptible to ADM-FSGS than any other strain, suggesting that the tendency to Th2 responses favors ADM-induced nephrology. We have shown here that, together with a Th2-like profile represented by high levels of GATA3, IL-4 and eotaxin, ADM-treated mice produced a mix of inflammatory cytokines from both the innate and Th17 “arms” of the immune system that were inhibited by GSL-1 treatment. Therefore, our data indicate that the Th1/Th2 imbalance may be simply one aspect of a more intricate mechanism of glomerular injury.

Despite the association between Th2 cytokines and FSGS, the mechanism involved in podocyte susceptibility to ADM cytotoxicity is not fully understood. Holthöfer et al. showed that podocyte injury was associated with a decrease in glomerular disialogangliosides (GD3) in a model of puromycin induced-FSGS [30]. Simons et al. demonstrated that lipid rafts containing GD3 gangliosides are involved in podocyte foot process effacement, indicating an important role for raft components in both pathological and physiological processes [32]. It has also been shown that the expression of GM3, the GD3 precursor, inhibited ADM-induced Lewis cell carcinoma death due to the induction of the anti-apoptotic protein Bcl-2 [33]. Therefore, gangliosides can be associated with both the conservation of podocyte structure and resistance to ADM cytotoxicity. In this regard, we found that ADM administration decreased the mRNA levels of GD3 synthase (ST8Sia1) when compared with control mice, suggesting the inhibition of its transcription. These data could reflect a decrease in the presence of GD3 within rafts and thereby explain podocyte injury. Consistent with this idea, GSL-1 treatment increased both GD3 synthase and GM3 synthase transcripts in association with an increase in the mRNA levels of the anti-apoptotic protein Bcl-2. Therefore, our data suggest that iNKT activation induces the metabolism of endogenous glycolipids that can promote either the preservation of podocyte structure due to GD3 conservation or resistance to ADM-induced cytotoxicity via the GM3/Bcl-2 axis. The induction of endogenous gangliosides by GSL-1 treatment is consistent with previous studies showing an association between iNKT cell activation and glycolipid metabolism [27], [35].

In addition to a Th2-like profile and changes in ganglioside metabolism, the pathogenesis of glomerulosclerosis is also marked by the presence of the fibrogenic cytokine TGF-β, which has been implicated in podocyte apoptosis, proliferation, epithelial-to-mesenchymal transition and glomerular matrix deposition [23], [36], [37], [38]. We demonstrated in an anti-glomerular basement membrane model of glomerulonephritis that iNKT cells modulate their pathogenesis through an alternative TGF-β signaling pathway, indicating a renoprotective role for TGF-β-iNKT axis and TGF-β-induced genes (TGFBI/BIGH3) [26]. Consistent with these results, we found that iNKT activation through GSL-1 treatment increased the TGF-β protein levels in serum and the expression of both TGF-β and TGFBI transcripts in kidney compared with both the control and ADM groups. These data corroborate the idea that the renoprotective effect of iNKT activation is somehow associated with TGFBI expression and suggest that TGF-β signaling differs between ADM and ADM+GSL-1 animals. In fact, ADM administration resulted in an increase in the expression of phosphorylated SMAD2/3, whereas GSL-1 treatment augmented the levels of the inhibitory SMAD7 protein.

The role of TGFBI protein in the kidney still remains unclear, but TGFBI is part of the extracellular matrix and has been implicated in the regeneration of renal proximal tubular epithelia, consistent with a renoprotective function [39]. Regarding the SMAD7 protein, Shiffer et al. suggested that it is activated during podocyte injury to control TGF-β/SMAD signaling and restore kidney physiology [40]. In the same context, Wang et al. demonstrated that in response to latent TGF-β1 signaling, SMAD7 inhibits NF-κB-driven inflammatory responses [41]. Thus, SMAD7 augmentation in response to GSL-1 treatment is consistent with the ideas that this protein is associated with podocyte and renal physiology and that GSL1 can modulate TGF-β signaling.

In conclusion, our work demonstrates that FSGS pathogenesis is regulated by an intricate network involving different inflammatory cytokines and chemokines, glycolipid metabolism and TGF-β signaling. We have also shown that pro-Th1 iNKT agonists can modulate this network to maintain podocyte physiology, suggesting a new approach to FSGS management.

## Materials and Methods

### Ethical Statements

The animals used in this work were housed in individual standard cages and maintained on a 12-h light/dark cycle in a temperature-controlled room at 21–23°C with free access to water and food. All procedures were approved by the internal ethics committee of the Federal University of São Paulo (1874/07).

### Animals and treatments

Isogenic male Balb/c mice, aged 8–12 weeks (23–28 g), were obtained from the Animal Care Facility at the Federal University of São Paulo (UNIFESP). The BALB/c Jalpha18^−/−^ mice were a gift from Dr. Masaru Taniguchi at the RIKEN Research Center for Allergy and Immunology (Japan) [17]. All animals were housed in individual standard cages and had free access to food and water. All procedures were previously reviewed and approved by the internal ethics committee of the institution. Focal segmental glomerulosclerosis was induced in mice using a single tail-vein injection of 10 mg/kg adriamycin (Doxorubicin hydrochloride, Pfizer, NY, USA); an equal volume of saline was given to control mice [15], [42], [43]. GSL-1 (5 µg/animal i.v.) was administered concomitantly with the ADM administration or four days later. GSL-1 glycolipid was synthesized as previously described [13].

### Renal function

To evaluate the renal function of the mice, urine samples were collected at different time points to quantify proteinuria and the albuminuia∶creatininuria ratio. All samples were analyzed using commercially available colorimetric assays: Labtest (Minas Gerais, Brazil) for creatinine measurements and Sensiprot® (Minas Gerais, Brazil) for protein measurements. To estimate the urinary albumin concentration, 10 µL of urine (1 mg/mL), corrected for the urinary creatinine levels, was run on a 10% SDS-PAGE gel and Coomassie stained. The densities of the bands present in the gel were analyzed using the GeneSnap and Gene Tools software (Syngene, UK).

### Real-time PCR analysis

On the day of sacrifice, kidney samples were quickly frozen in liquid nitrogen. Total RNA was isolated using the TRIzol Reagent (Invitrogen, USA). First-strand cDNAs were synthesized using the MML-V reverse transcriptase (Promega, USA). Real-time PCR was performed using the TaqMan PCR assay and the following probes: TNF-α, Mm00443258; PAI-1, Mm 01204469; vimentin, Mm00801666; BCL-2, Mm 02528810; MMP9, Mm01240560 (Applied Biosystems, USA). For the analyses of IL-4, IL-13, GATA3, T-bet, glycosyltransferases, TGF-β and TGFBI (BiGH3), real-time PCR was performed using SYBR Green (Table 1) (Applied Biosystems, USA). The cycling conditions used with the Taqman and SYBR Green primers were as follows: 10 min at 95°C, followed by 45 cycles of 30 s at 95°C, 30 s at 60°C and 30 s at 72°C. The relative quantification of mRNA levels was performed using the comparative threshold cycle method (Applied Biosystems, USA). Briefly, the target gene amount was normalized to the endogenous reference gene (HPRT, SYBR Green), and then normalized to a calibrator (control animals) using the formula 2^−ΔΔCt^. Thus, all data were expressed as an N-fold difference related to the expression in the matched controls. Analyses were performed using the Sequence Detection Software 1.9 (Applied Biosystems, USA).

**Table 1:** Primer sequences for mRNA quantification.

Gene	Sense	Antisense
**HPRT**	5′CTCATGGACTGATTATGGACAGGAC3′	5′GCAGGTCAGCAAAGAACTTATAGCC3′
**St3Gal5 (GM3synthase)**	5′GCGAAGACGGCTATGGCTCT3′	5′ TCCGGAATCCAAAAGGCG 3′
**ST8Sia1 (GD3synthase)**	5′CCTTCCAGCTGCCATTGAAG3′	5′GAATCCCACCGTTTCCCAC3′
**Gata-3**	5′AGAACCGGCCCCTTATCAA3′	5′ AGTTCGCGCAGGATGTCC 3′
**T-bet**	5′CAACAACCCCTTTGCCAAAG3′	5′ TCCCCCAAGCAGTTGACAGT 3′
**TIMP-1**	5′ACAGGAGAAGGGACGCCATG3′	5′ GCAGCTTATCGATGAATCCA 3′
**IL-4**	5′ACAGGAGAAGGGACGCCATG3′	5′GCAGCTTATCGATGAATCCCA3′
**IL-13**	5′GCTTATTGAGGAGCTGAGCAACA3′	5′ GGCCAGGTCCACACTCCATA 3′
**CXCL16**	5′TGAACTAGTGGACTGCTTTGAGC3′	5′GCAAATGTTTTTGGTGGTGA3′
**TGF-β**	5′TGGAGCAACATGTGGAACTC3′	5′GTCAGCAGCCGGTTACCA3′
**TGFBI**	5′TCCTTGCCTGCGGAAGTG3′	5′GGAGAGCATTGAGCAGTTCGA3′

### Renal histology analysis

On the day of sacrifice, kidneys were fixed in 10% neutral formalin for 24 h and then embedded in paraffin. Sections (3 µm) were stained with hematoxylin/eosin and analyzed using a trinocular optical microscope (Olympus Corporation, Japan). Glomerulosclerosis was evaluated based on the percentage of glomeruli damaged [44]. Tubulointerstitial injury was defined as tubular dilation and/or atrophy or characterized by interstitial fibrosis, as previously described [45], [46]. Tubular injury was scored as follows: 0 = changes in <10% of the cortex; 1+ = changes in up to 25% of the cortex; 2+ = changes in up to 50% of the cortex; and 3+ = changes in >50% of the cortex sections.

TGFβ-1 protein levels were measured by enzyme-linked immunosorbent assay (ELISA).

TGFβ-1 protein was assessed in kidney tissues lysed with RIPA Buffer (25 mM Tris•HCl pH 7.6, 150 mM NaCl, 1% NP-40, 1% sodium deoxycholate, 0.1% SDS) supplemented with a protease inhibitor (Sigma Aldrich, USA). Total TGFβ-1 protein was measured using a TGFβ-1 Emax immunoassay system (Promega, USA) according to the manufacturer's instructions. The results were presented as pg of TGFβ-1/µg of total protein measured using the Bradford assay (Bio-Rad, USA).

### Western blot analysis

Briefly, 100 µg of total protein from renal tissue were collected and then diluted in sample buffer (Bio-Rad, USA) containing 20 mg/ml of 2-β- mercaptoethanol (Sigma, United States). The samples were denatured for 5 min at 95°C and then separated on a 10% polyacrylamide electrophoresis gel. Next, the proteins were transferred onto a nitrocellulose membrane, blocked for an hour with 5% albumin diluted in TBS-T solution and then incubated with the primary antibody diluted in TBS. Finally, the membrane was washed with TBS for an hour and incubated with the secondary biotinylated antibody. The molecular masses of the proteins were determined by comparison with the migration of rainbow markers (Bio-Rad, USA). The following antibodies were used for western blotting: phosphorylated SMAD2/3, SMAD7 and GAPDH (Santa Cruz Biotechnology Inc., USA) and MMP9, PAI-1, Desmin and HPRT (Abcam, USA).

### Statistical analysis

All data are presented as the mean ± SEM. Different results among the groups were compared by ANOVA. The threshold for significance was established as p<0.05. All statistical analyses were performed with the aid of GraphPad PRISM (Graphpad, USA).

## Supporting Information

Figure S1 **GSL-1 treatment inhibits the expression of fibrogenic proteins.** BALB/c mice were injected at day 0 with 10 mg/kg of adriamycin (ADM) or treated concomitantly with ADM and 5 µg/mouse GSL-1 (ADM+GSL-1). Consistent with the transcript analysis of kidney tissue, ADM mice showed a slight increase in the expression of PAI-1 protein (B), without significant alteration in MMP9 protein levels (A), when compared with the control and ADM+GSL-1 animals. In contrast, Desmin expression significantly increased in ADM mice when compared with both the control and GSL-1-treated groups (C). These data corroborate our previous mRNA analysis and further demonstrate the protective effect of GSL-1 treatment.
(TIF)
