# Supplementary figures and images for: Invariant Natural Killer T Cell Agonist Modulates Experimental Focal and Segmental Glomerulosclerosis
Source: PLoS One. 2012 Mar 12;7(3):e32454. doi: 10.1371/journal.pone.0032454 (PMC3299669; doi:10.1371/journal.pone.0032454)

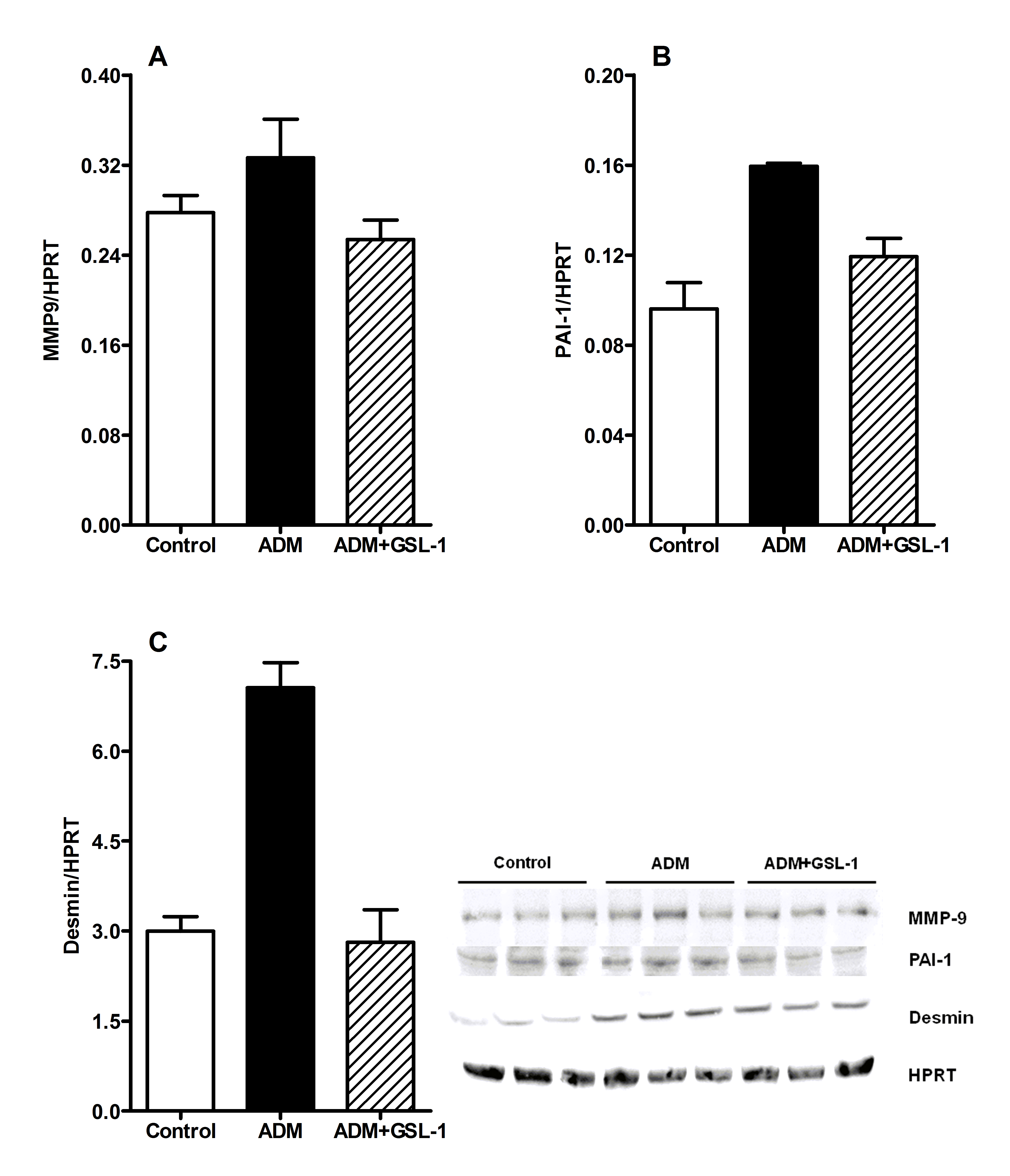

Supplement: Figure S1 — GSL-1 treatment inhibits the expression of fibrogenic proteins. BALB/c mice were injected at day 0 with 10 mg/kg of adriamycin (ADM) or treated concomitantly with ADM and 5 µg/mouse GSL-1 (ADM+GSL-1). Consistent with the transcript analysis of kidney tissue, ADM mice showed a slight increase in the expression of PAI-1 protein (B), without significant alteration in MMP9 protein levels (A), when compared with the control and ADM+GSL-1 animals. In contrast, Desmin expression significantly increased in ADM mice when compared with both the control and GSL-1-treated groups (C). These data corroborate our previous mRNA analysis and further demonstrate the protective effect of GSL-1 treatment. (TIF) [file pone.0032454.s001.tif]
